# Self-Care Behaviors of Patients With Heart Failure in Thailand: A qualitative descriptive study

**DOI:** 10.1177/10436596251323269

**Published:** 2025-03-14

**Authors:** Apinya Koontalay, Mari Botti, Anastasia Hutchinson

**Affiliations:** 1 School of Nursing and Midwifery, Faculty of Health, Deakin University, Geelong, Victoria, Australia; 2 Center for Quality and Patient Safety Research-Epworth HealthCare Partnership, Deakin University, Geelong, Victoria, Australia

**Keywords:** heart failure, climate, consumer experience, qualitative research, self-management

## Abstract

**Introduction:**

This study explored people’s experiences of living with heart failure and their understanding of self-management and difficulties faced when making lifestyle changes in the context of high-salt food preferences and a subtropical climate.

**Methods:**

This qualitative descriptive study was conducted with 20 Thai individuals with heart failure. Data were analyzed using thematic content analysis.

**Results:**

Two overarching themes were: (a) adapting long-established dietary and lifestyle behaviors is challenging in the context of limited knowledge of heart failure, cultural food preparation practices and a subtropical climate and (b) personal values, attitudes, and preferences have primacy over dietary and fluid restrictions.

**Discussion:**

In Thailand, strong cultural preferences for high salt, preprepared street foods contribute to repeated admissions for decompensated heart failure. Community-based models of care are urgently needed that support effective chronic heart failure management, with solutions that consider local needs, climate and cultural factors.

## Introduction

People with heart failure (HF) experience symptoms that affect their health-related quality of life (Santos et al., 2021). Lifelong adherence to self-care management is required for condition stability (Aghajanloo et al., 2021). Nonadherence to HF self-care is associated with poorer outcomes, including higher 30-day readmission rates (Madanat et al., 2021), higher mortality (Savarese et al., 2022), and lower quality of life (Van Spall et al., 2017). Worldwide, approximately 64.3 million people have HF, with an estimated nine million living in Southeast Asia (*SE*-Asia) and approximately 2.8 million in Thailand (Lam, 2015; Savarese et al., 2022). The prevalence of HF is increasing in many low- and middle-income countries (LMICs) because of increasing life expectancy, urbanization, and changes in lifestyle (Ang et al., 2023; Savarese et al., 2022). Maintaining treatment and lifestyle adjustments is challenging for patients and their families (McDonagh et al., 2022).

HF management involves pharmacological and nonpharmacological treatments (Heidenreich et al., 2022; McDonagh et al., 2022). Currently, evidence-based nonpharmacological treatments are dietary, specifically avoidance of excessive sodium and fluids (Camafort et al., 2023; Heidenreich et al., 2022; McDonagh et al., 2022). However, there are not unified guidelines for HF in south-east Asia; each country has its own guidelines (Asia-Pacific Cardiovascular Disease Alliance, 2024) and only a few countries have dedicated HF specialists. Climate, culture, and dietary practices and preferences, coupled with unequal access to health services and limited resources, present unique challenges for people living with HF in LMICs (Ang et al., 2023; Koontalay et al., 2024a). These challenges influence adherence to lifestyle recommendations (Lam, 2015). Associations between acute hospital admissions for HF-related fluid overload and variability in daily temperature have been shown (Gasparrini et al., 2017; Vishram-Nielsen et al., 2023).

Climate can increase the burden of heart disease and influence the type and severity of symptoms (Vishram-Nielsen et al., 2023). In countries in *SE*-Asia such as Thailand, hot and humid conditions prevail, and daily peak temperatures over 30°C persist for more than 6 months of the year (The World Bank Group and the Asian Development Bank, 2021), impacting on cardiovascular disease-related mortality (Vishram-Nielsen et al., 2023). Alahmad et al. (2023) investigated the association between extreme temperatures and cardiovascular-specific mortality in 27 countries and found that between 1999 and 2008, 55 cities across Thailand had the highest HF burden and cardiovascular diseases (CVDs)-related mortality due to extremely high temperatures (Alahmad et al., 2023). These climatic conditions influence body temperature regulation, hydration and metabolism, leading to increased thirst, high fluid intake and salt depletion (Jacobsen et al., 2022; Periard et al., 2021; Vishram-Nielsen et al., 2023).

Although restriction of salt and fluids are considered beneficial and well-established recommendations for individuals living with HF, the assumption of universal positive impacts is now being questioned (Jaarsma et al., 2021). One recent systematic review and meta-analysis found evidence that sodium restriction may increase mortality and rehospitalisation, while restricting fluid intake had a beneficial effect on these outcomes (Li et al., 2022). Other recent systematic reviews found that sodium restriction while not harmful did not decrease risks of readmission or mortality (Colin-Ramirez et al., 2022; Stein et al., 2022). The potential for routine guidelines to be associated with undesired health outcomes highlights the importance of the context in which they are applied. HF lifestyle recommendations are based on Western dietary and cultural traditions that differ from those of Asian or *SE*-Asian cultures. Adaptation of HF programs to different cultural contexts is a global public health issue, and more evidence is needed to provide supportive care that meets patients’ needs. The purpose of this study was to explore people’s experiences of living with HF, their understanding of HF self-management, and the challenges they face when making recommended lifestyle changes.

## Method

### Study Design

A qualitative descriptive, cross-sectional study design with individual semi-structured interviews was used. Qualitative description is a methodological approach intended to stay as close to the data as possible (Sandelowski, 2009). This design was considered appropriate to our aim of exploring people’s experiences of living with Heart Failure and their understanding of self-management and difficulties faced when making lifestyle changes in the context of high-salt food preferences and a subtropical climate. The consolidated criteria for reporting qualitative research (COREQ) checklist was used to report the findings (Tong et al., 2007).

### Study Setting

The study was conducted in a hospital in Thailand chosen based on its size and central location in Bangkok. The tertiary referral hospital (500 beds) receives patients from a wide area in Thailand, including urban and rural regions. The HF follow-up program was limited to one outpatient clinic visit 2 weeks after discharge.

### Participants and Recruitment

Participants were recruited by purposive sampling methods. Inclusion criteria were: (a) aged 18 years or above; (b) a confirmed diagnosis of HF for at least 6 months, (c) current hospitalization with an exacerbation of HF, and (d) at least one other acute care presentation (ED visit or admission) with an episode of acute decompensated HF within the previous year. Patients were excluded if unable to converse in Thai, provide informed consent or participate in an interview because of delirium, dementia or other cognitive impairment. The eligibility criteria were explained to a cardiac nurse at the hospital who then identified potential participants, informed them of the study and asked their permission to provide their names to the primary researcher, who although affiliated with the hospital did not have a clinical role. This researcher then visited potential participants to obtain consent and arrange a convenient time for the interview prior to their discharge. All participants approached by the researcher agreed to participate. We initially planned to interview 12 to 15 patients, but continued until data saturation was reached (*n* = 20; Guest et al., 2020).

### Ethical Considerations

This study was approved by the research and ethics committees of the hospital in Bangkok, Thailand (LH631036) and Deakin University (HEAG-H 39-2021). Written informed consent was obtained from participants.

### Data Collection

Face-to-face semistructured interviews were conducted between February and May 2021. The interviews were conducted in Thai by AK, a bilingual Thai national, doctoral student and registered nurse who received training and supervision in qualitative research methods in her doctoral studies. Participants were interviewed at the bedside prior to discharge from the hospital. A semistructured interview guide was used to ensure consistency in questioning but also allow participants to express their thoughts in their own words and expand on issues raised (Gerson & Damaske, 2020). The interview guide was derived from the literature on the experiences of living with HF (Table 1). Probing questions were used once participants responded to open-ended questions designed to gain insights into their experiences of managing their lifestyles and symptoms and the challenges associated with HF. To build rapport, interviews began by gathering demographic and background information, which included exploring their cultural context, home language and social supports. The overall approach provided valuable content and rich descriptions for data analysis. The audio-recorded individual interviews lasted 30 to 45 minutes. Participants were given the opportunity to postpone the interview if they felt unwell or did not want to continue. This did not occur.

**Table 1.** Interview Guide: Topics, Open-Ended Questions and Probes.

Topic	Description	Open questions	Example probes
Self-management in everyday life	Patients’ activities of daily living to manage HF with a focus on dietary and fluid restrictions.	Can you describe a typical day in managing your heart condition?	Diet Can you describe what types of food you like? Who prepares the food that you eat? Can you describe what you think about when you shop for food? Fluids Do you know how much you weigh? How often do you weigh yourself? How do you know how much fluid you drink in a day? How do you manage your medications to accommodate your HF in your everyday life?
Challenges associated with self-management of HF	The impact of HF on everyday activities and the challenges faced in adapting and managing dietary and fluid restrictions.	Many people find it difficult to limit how much they drink each day and reduce salt in their diet.—Can you describe how these changes or restrictions affect you?	How has your lifestyle changed (in terms of medications, diet, and daily activities) compared to before your diagnosis? What resources do you use to help you make changes to your diet and fluids? What sorts of things interfere with your ability to manage your HF?

### Data Analysis

Audio recordings were transcribed checked for accuracy and translated, into English for later analysis. To ensure accuracy and authenticity of the English translation, both versions of the transcripts (Thai and English) were reviewed by a professional language expert based in Thailand. English translations were refined until the language expert agreed that they were accurate reflections of the original Thai versions. Data were analyzed using thematic content analysis based on the processes summarized by Kiger and Varpio (2020). The first step was data familiarization where transcripts were read through several times to obtain a context-based understanding of the data. This was followed by a combination of deductive and inductive approaches to the analysis. Initial codes were generated by reading the raw data line by line and using margin notations and labels. These initial codes were then grouped into the content categories of the interview topics, that is, self-management of diet and fluids and challenges to self-management. This enabled the identification of subcategories within these topic areas. The next step was to generate themes and subthemes by searching for unifying patterns in the data. Transcripts were then re-read to ensure that data fit was meaningful. The final step was to name and define the themes.

### Rigor

Rigor was considered in several ways. All interviews were conducted by the same interviewer using an interview guide. To maintain the trustworthiness of the analysis, all research team members, including the doctoral committee members and were actively involved in all aspects the analysis and interpretation. The first author, the interviewer, performed the initial sorting and coding of data. The research team, which included experienced qualitative researchers, independently reviewed the interview transcripts, discussed codes, themes and subthemes and confirmed representative transcribed quotations in relation to themes. Agreement of the final themes and descriptions was determined through discussion and review of the data. The raw data and coding documents provide evidence of each step in the analytical process. Data saturation was determined by reviewing interview audiotapes in cycles of four interviews. It was not logistically possible to contact participants to establish confirmability of the analysis once they had left the hospital.

## Results

### Participants Characteristics

Twenty patients were interviewed; saturation occurred after the fourth cycle (16 patients). The age range of participants was 42 to 78 years. The duration of HF ranged from one to 30 years, and severity of illness, according to the New York Heart Association Classification, varied (Table 2). Thirteen patients had been admitted to the hospital at least three times in the past year, duration of HF ranged from 1 to 5 years. Two patients were unable to read or sign consent forms because of illiteracy and used thumbprints to provide consent.

**Table 2.** Characteristics of Participants (N = 20).

Characteristics	Patients
Age group (years)	
41–50	8 (40%)
51–60	5 (25%)
61–70	3 (15%)
≥71	4 (20%)
Sex	
Male	16 (80%)
Female	4 (20%)
The New York Heart Association classification	
Class II	10 (50%)
Class III	9 (45%)
Class IV	1 (5%)
Length of time with CHF (years)	
1–5	12 (60%)
6–10	5 (25%)
11–15	1 (5%)
≥16	2 (10%)
Reattendance experience within the previous year (times)	
1–3	13 (65%)
4–6	5 (25%)
≥7	2 (10%)

### Overview of Findings

Participants described their experiences of making adjustments to dietary and fluid recommended restrictions. The themes that emerged reveal the complex interplay of factors associated with self-care behaviors in this context. Two overarching themes were: (a) Adapting long-established dietary and lifestyle behaviors is challenging in the context of limited knowledge of HF, cultural food preparation practices and a subtropical climate, and (b) Personal values, attitudes, and preferences have primacy over recommended dietary and fluid restrictions.

### Theme 1: Adapting Long-Established Dietary and Lifestyle Behaviors Is Challenging in the Context of Limited Knowledge of HF, Cultural Food Preparation Practices and a Subtropical Climate

Participants described the many factors that challenged their ability to comply with dietary and fluid guidelines. What was most apparent was the lack of strategies to cope with the effects of climate and cultural food acquisition practices. One of the main impediments to fluid restrictions was the effect of climate.

### Climate and Thirst

Most participants found it challenging to comply with the fluid intake restrictions of 1.5 to 2 liters per day during hot and humid weather. They recognized that fluid overload caused HF exacerbations but had no strategies for managing restrictions.

The doctor said the symptoms had worsened this time [required intensive care and mechanical ventilation this admission] because I had been drinking too much water. It is difficult to control fluids because our home is hot, making me feel thirsty. What am I supposed to do when I’m thirsty? (ID: 12, HF duration: 2 years, female, age: 48 years)

Interestingly, many participants preferred to hydrate with sugary beverages to reduce their thirst.

I like to drink cold water, as it refreshes me. I usually have a little bucket [2.5 L] of cold Pepsi everywhere I go. It is hot and that makes me thirsty and moody. So, the cold drink makes me feel better and calms me. (ID: 9, HF duration: 27 years, female, age: 74 years)

### Food Preparation and Acquisition

Cultural food preparation practices impacted participants’ ability to adopt new dietary patterns and adhere to a low-salt diet. These practices included food preparation and food sharing. In many cases, participants did not have control over their diet because family members were responsible for food preparation:

It is not easy to reduce salt because my sister cooks for everyone in my family, and I have to eat what she cooks. She usually puts MSG in every dish, and I try to reduce the sauce or MSG, but I cannot live without it. (ID: 7, HF duration: 6 years, male, age: 46 years)

Reliance on street food without nutritional labels was very common. Although some participants recognized the issues related to consumption of street food, their family members may not have been aware that these foods may not meet the dietary recommendations for people with HF. There were attempts by some participants to alter high salt dishes:

My daughter occasionally buys Isan [north-eastern] food from a local market, where they use a lot of MSG and fermented fish, such as curry chicken with dill or bamboo shoot curry. I told my daughter to add more water to dilute it if it’s too salty. I knew the salty taste would make me thirsty, making be drink a lot of water. (ID: 9, HF duration: 27 years, female, age: 74 years)

### Knowledge of Heart Failure and Self-Care Resources and Strategies

Limited understanding of the disease and failure to recognize the significance of HF symptoms compounded the effects of climate and food acquisition and preparation. There was sometimes confusion about dietary restrictions:

I thought eating spicy or salty foods could help me get rid of anorexia. I was wrong because spicy or salty [food] made me thirstier, and then I was swollen and had difficulty breathing later.” (ID: 8, HF duration: 30 years, male, age: 71 years)

Also, most participants did not monitor their weight and were unaware that sudden weight gain could be a sign of HF progression:

I have never weighed myself at home. I was weighed when I was in hospital for check-ups. I do not have scales at home. (ID: 17, HF duration: 2 years, male, age: 48 years)

One participant when asked about self-adjustment of diuretics based on weight fluctuations responded:

I have never weighed myself because I do not have scales at home. I never adjust my medications; I have never taken extra diuretics when I have a flare-up. I only take medicines prescribed by the doctor. (ID: 9, HF duration: 27 years, female, age: 74 years)

Some participants however, who recognized the relationship between worsening symptoms and self-care would modify behaviors in response to signs of deterioration:

Whenever the swelling begins in both legs and then in my testicles. . . when symptoms such as tiredness or a slight swelling in my legs appear, I realise I need to reduce my water intake slightly. (ID: 7, HF duration: 6 years, male, age: 46 years)

Participants described limited access to community disease management resources following discharge from hospital and had few strategies to help them shift away from their traditional dietary preferences and adopt new dietary patterns. Overall, they lacked the knowledge and support necessary to self-manage their disease and avoid unnecessary hospitalisations. In terms of diet, participants simply did not know how to make changes that would be palatable and in keeping with their preferred comfort foods. For example, one participant noted the difference between the low-salt meals served in the hospital and the preferred meals eaten at home:

Now that I’m readmitted to the hospital; I see the food here has no flavour and is bland because the doctor has [recommended] limited salt and water. It is completely different in taste compared to my foods at home. (ID: 8, HF duration: 30 years, male, age: 71 years)

Participants appeared to have received very little information about diet:

The doctor says to me ‘don’t forget diet control.’ But I don’t know what food she recommends. I know that diet control is important, but what should I avoid? . . .I didn’t know and I was scared to ask the doctor. . .every doctor says the same thing: control your diet [laughs]. (ID: 13, HF duration: 2 years, male, age: 54 years)

### Theme 2: Personal Values, Attitudes, and Preferences Have Primacy Over Recommended Dietary and Fluid Restrictions

Although knowledge and food acquisition and preparation were important challenges, dietary attitudes, values and preferences also affected participants’ food choices and their willingness to change their eating habits, making it difficult for them to adjust to a low-salt diet. Most participants, although aware of the need to reduce their salt intake, described their preferences for traditional Thai foods such as fermented fish, or salty seasonings such as fish sauce. Familiarity with particular foods also played a part in participants’ diets.

### Food Preferences and Practices

Long-standing dietary habits and preferences for Isan or north-eastern traditional Thai street foods was common made it challenging for participants to make alternative dietary choices:

I like to eat papaya salad, a spicy sauce dip with fermented fish, or anything from the north-east, such as raw pork called Lab Mao. I usually buy my food from the street market because it is convenient and offers many different types of foods, and it is cheap.” (ID: 13, HF duration: 2 years, male, age: 54 years)

This preference for street food was often described by participants:

I like to buy food from the market such as green curry, sour soup, or traditional Thai desserts such as golden thread, golden drop, and pandan coconut custard and noodles every day. (ID: 1, HF duration: 2 years, male, age: 68 years)

In addition to street foods, adding salt or seasoning, such as fish sauce or MSG at the table or during cooking was also common:

I always add chilli with fish sauce to my plate because I am a salty person [laughing]. My foods need to have added MSG or fish sauce. (ID: 7, HF duration: 6 years, male, age: 46 years)

These food choices persisted even when facing symptoms of acute HF deterioration:

Even when I was tired and had trouble breathing, I still ate papaya salad with fermented fish or would add fermented fish to my soup every day. I love it and I can’t live without it. (ID: 13, HF duration: 2 years, male, age: 54 years)

### Expectations of Failure to Change

Prominent among participants was a sense of frustration and anticipation of failure associated with dietary and fluid management of HF.

[smiles] Every time I go back home after being in hospital for a few days, I think I can control myself by restricting fluids and salt. In reality, I fail so many times. . . I can’t do it. (ID: 7, HF duration: 6 years, male, age: 46 years)

The expectation of not being able to restrict fluids for example, further illustrated the lack of strategies these participants had to be successful in making changes to long-standing behaviors:

If I get home this time, I think I can control myself. Sometimes I accidentally drink a lot of water. . .but mostly [previously] I was out of control because of non-compliance with self-care. I knew everything but I couldn’t do it [smiles]. (ID: 8, HF duration: 30 years, male, age: 71 years)

## Discussion

This study aimed to gain insight into the experiences of individuals with HF living in a subtropical climate. Cultural preferences, knowledge, social norms, and environmental contexts impacted participants’ health behaviors with significant consequences in terms of frequent readmissions to the hospital, sometimes with severe decompensated HF. Climate, especially during the extreme heat and humidity of a subtropical summer, contributed to participants’ low levels of self-reported adherence to lifestyle modification strategies such as fluid and salt restriction. This was compounded by limited understanding of the pathophysiology of HF, strong personal preferences for traditional Thai dishes high in salt, ease of access to cheap, street foods without nutritional labeling, and cultural patterns of communal food preparation. Overall, participants had very few strategies and little support for making adaptations to their dietary and fluid consumption.

Traditional salted and fermented foods and condiments are an integral part of *SE*-Asian cuisine and contribute to the high sodium intake in these countries (Amarra & Khor, 2015; Silva-Santos et al., 2021). In these regions, meals tend to be prepared for the whole family, and people are often unaware of their estimated salt intake. This poses a challenge to health care providers who educate patients and their families about the effects of high sodium diets to develop approaches that empower and motivate individuals with HF to make healthier dietary choices. Most Thai participants with HF expressed their preference for ready-made one-plate meals or eating outside the home, substantially affecting compliance with dietary recommendations.

Lack of community disease management support once discharged from the hospital compounded issues related to food preferences. High sodium intake is culturally and regionally specific in Asia, especially in Thailand (Chailimpamontree et al., 2021), where high-sodium dietary components such as monosodium glutamate (MSG), fish sauce, and soy sauce are common (Amarra & Khor, 2015; Chailimpamontree et al., 2021). Consequently, average sodium consumption in most Asian countries is higher than recommended by the World Health Organization (WHO). Estimates are that daily sodium consumption in Thailand is twice the WHO-recommended intake (Chailimpamontree et al., 2021). Whether salt-restricted diets in the context of HF (i.e., diets with salt intake below recommendations for the general population) are beneficial is inconclusive (Colin-Ramirez et al., 2022; World Health Organization [WHO], 2023); however, there is substantial evidence that a relative reduction in mean salt intake of 30% would reduce global burden of disease (WHO, 2023). Providing social support and family training programs on healthy lifestyle behaviors may increase knowledge, promote self-efficacy, and support personal care benefiting long-term care of people in the community with chronic health conditions (Chiaranai et al., 2018). Resources are needed that support patients and families to maintain low-salt diets in contexts where nutritional labels are not available and that accommodate food preferences, in particular when these preferences take priority over dietary recommendations.

The importance of adapting lifestyle recommendations to the local context is particularly relevant to fluid intake guidelines for people with HF (Jaarsma et al., 2021). Our findings are consistent with those of several studies exploring the experiences of people with HF in tropical or extremely hot climates, such as in Thailand, Singapore, and Vietnam where recommendations can sometimes be inflexible (Khankaew et al., 2020; Phung et al., 2016; Rong et al., 2017; Seah et al., 2016). According to International HF guidelines, such as the European Society of Cardiology (ESC) guidelines, fluid intake recommendations should be individualized by increasing recommended fluid intake during periods of high heat and humidity and closely monitoring body weight (McDonagh et al., 2022). People with severe HF are advised to monitor and limit their fluid intake to 1,500 to 2,000 mL/day, whereas no restrictions are recommended for those with mild or moderate symptoms (Heidenreich et al., 2022; McDonagh et al., 2022). Fluid intake advice should also consider differences in body weight between individuals with HF (Jaarsma et al., 2021).

In addition to the need for flexibility in lifestyle recommendations, international HF programs and guidelines based on standardized practices developed in Western countries may need to be adapted before being integrated into practice in tropical areas, where excessive fluid loss and thirst are likely. They may not be directly applicable to certain regions, such as South-East Asia, due to differences in culture, environment, patient self-efficacy, and available resources (Koontalay et al., 2024b). Most participants in our study needed support to gain a better understanding of self-care recommendations and none were routinely monitoring weight fluctuations. Engagement of patients and their families in care planning and self-management activities was limited due to the brief postdischarge follow-up period of 2 weeks. Most participants indicated they needed greater ongoing support to monitor and manage their symptoms and all struggled to adhere to dietary and fluid recommendations. The findings underscore the need for health care providers to incorporate a more nuanced approach to fluid management and develop proactive strategies to help patients adapt and sustain these changes.

### Implications for Practice

Our findings highlight the need for formative interventions to support lifestyle modifications for individuals with HF who live in tropical climates and represent diverse cultures. Such interventions would include education programs on how to implement lifestyle changes, reduce high-risk behaviors, and make healthier dietary choices (Tariq et al., 2022; Vos et al., 2022). It is challenging to instill an understanding and awareness of the importance of nutritional labels in a context in which cultural eating habits are primarily focused on consumption of ready-made food from local markets or street foods, for which nutritional information is unavailable. Multidisciplinary, community-based salt reduction education programs are needed to address the factors that influence and motivate successful modification of eating behaviors (Silva-Santos et al., 2021). Public health policies are need to drive developments to implement population-wide salt reduction and healthier dietary behaviors (Jindarattanaporn et al., 2024).

The negative impact of heat on health, particularly for people with HF in LMICs, will continue to increase because of global warming, population growth, aging, and urbanization (Jacobsen et al., 2022). Understanding the lived experience of people living with HF can help inform national and international public health policies. Despite the known importance of reducing salt intake at a population level, the relative benefits of salt and fluid restrictions for individuals with HF living in hot and humid conditions with high insensible fluid losses has not been systematically evaluated. Disease management interventions should consider factors such as household and accommodation characteristics, behavioral patterns, and age where older adults have reduced thermoregulatory capacity (Jacobsen et al., 2022). In addition, the need for interventions to be linked to community services and incorporate seasonal climate conditions is evident (Alahmad et al., 2023; Irwan et al., 2022).

### Limitations and Strengths

This study highlights the importance of understanding climate-related challenges faced by people with HF living in Thailand. Interview questions were primarily focused on participants’ experiences of self-managing HF and it would have been useful to probe deeper into the strategies participants found practical and manageable when making recommended lifestyle modifications, particularly with limited resources and lack of ongoing support. We did not explore the impact of participants’ environmental conditions such as availability of climate control systems (e.g., air conditioning or natural ventilation) or socio-economic backgrounds that could have provided a richer understanding of how these factors might have impacted morbidity and general well-being and should be included in future research. The case study hospital was located in an urban area in Thailand with patients from both rural and urban areas, the findings however might not be transferable to more remote rural areas in Thailand. Purposive sampling of participants who had lived with HF for an extended period (e.g., five years or longer) and had experienced several recent exacerbations of their condition may limit transferability of the findings to individuals with more stable or controlled HF.

## Conclusion

Globally, the burden of a high salt intake constitutes a major public health challenge. For individuals living with HF in Thailand, where the preference for preprepared street foods impacts on individuals’ ability to adhere to a low salt diet, high salt consumption contributes to a pattern of repeated episodes of decompensated HF. The findings underscore the need for models of health service delivery to evolve to meet the needs of individuals living with HF in *SE*-Asia. Palatable alternatives to foods and condiments with high sodium content should be identified and changes made to the provision of patient and family education to include a tailored model of long-term care where patients are supported to develop their self-management skills and make lifestyle adjustments incrementally.
